# A large volume wind data for renewable energy applications

**DOI:** 10.1016/j.dib.2019.104291

**Published:** 2019-07-19

**Authors:** R. Bharani, A. Sivaprakasam

**Affiliations:** Department of Electrical and Electronics Engineering, Anna University, Chennai, 600025, India

**Keywords:** Wind speed, Wind direction, Wind power density, Wind energy potential, Geomorphology

## Abstract

The objective of the collection of dataset is to calculate the wind energy potential in the selected location using large volume of wind dataset. The wind energy potential data were collected at 100 m height from MSL (Mean Sea Level) from 2014 to 2016. The wind speed and direction were used to analyse wind energy characteristics and suitable site for wind turbine installation. The maximum wind power density was observed at monitoring sites S1, S2, S3 and S4. The altitude of the monitoring station and geomorphology of the site significantly controls the wind power density.

**Table:** Specifications Table

Subject	Renewable Energy, Sustainability and the Environment
Specific subject area	Application of Meteorological data in power production
Type of data	Table Figure
How data were acquired	Data acquired from Anemometer and Wind vane Instruments
Data format	Raw Analyzed
Parameters for data collection	Wind speed, Wind direction, Temperature, and Pressure data were collected at 100 m height from 12 different locations. The data is analyzed and compared for each location. Data processed using Microsoft Excel and prepared the Rose Diagram using MATLAB 2018b
Description of data collection	Wind speed is measured using anemometer and wind direction is measured using wind vane. The data are recorded for every 10 minutes throughout the year. In this analysis three years (2014, 2015, and 2016) of data are used for all 12 locations.
Data source location	Region: 12 data acquiring locations all along the Tamil Nadu Country: India Latitude and longitude for collected samples/data: S1 – Latitude: 08˚51′39.30″N and Longitude: 77˚53′11.40″E S2 – Latitude: 10˚34′33.20″N and Longitude: 77˚41′21.30″E S3 – Latitude: 10˚44′36.70″N and Longitude: 78˚08′17.00″E S4 – Latitude: 08˚57′44.05″N and Longitude: 77˚43′12.73″E S5 – Latitude: 10˚03′21.90″N and Longitude: 78˚42′46.00″E S6 – Latitude: 09˚04′47.50″N and Longitude: 78˚17′44.30″E S7 – Latitude: 11˚22′48.00″N and Longitude: 77˚10′50.70″E S8 – Latitude: 10˚08′28.20″N and Longitude: 77˚44′04.70″E S9 – Latitude: 09˚09′33.20″N and Longitude: 77˚31′46.70″E S10 – Latitude: 09˚28′23.10″N and Longitude: 77˚44′17.20″E S11 – Latitude: 11˚03′50.22″N and Longitude: 78˚39′04.90″E S12 – Latitude: 10˚38′17.74″N and Longitude: 78˚31′41.95″E
Data accessibility	With the article

**Table:** 

**Value of the Data** • The large volume of wind direction data can be used to analyse, the average annual wind direction of the site. • The wind data monitoring for large period was helpful to identify the suitable location for installing the wind turbines and also used to find the type of wind turbine. • The wind data based power density of the each site can be used to find the geological and geomorphological control on wind power generation.

## 1 Data

**Fig. 1:**
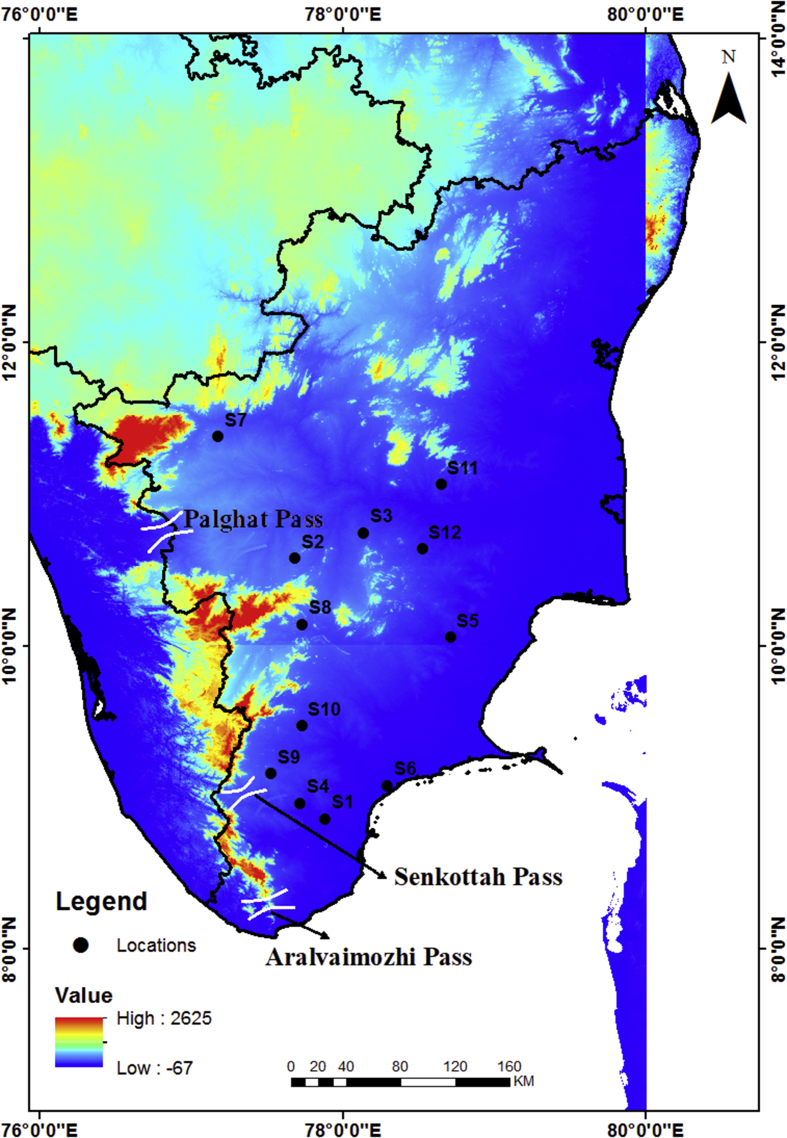
Location map with monitoring sites.

**Fig. 2:**
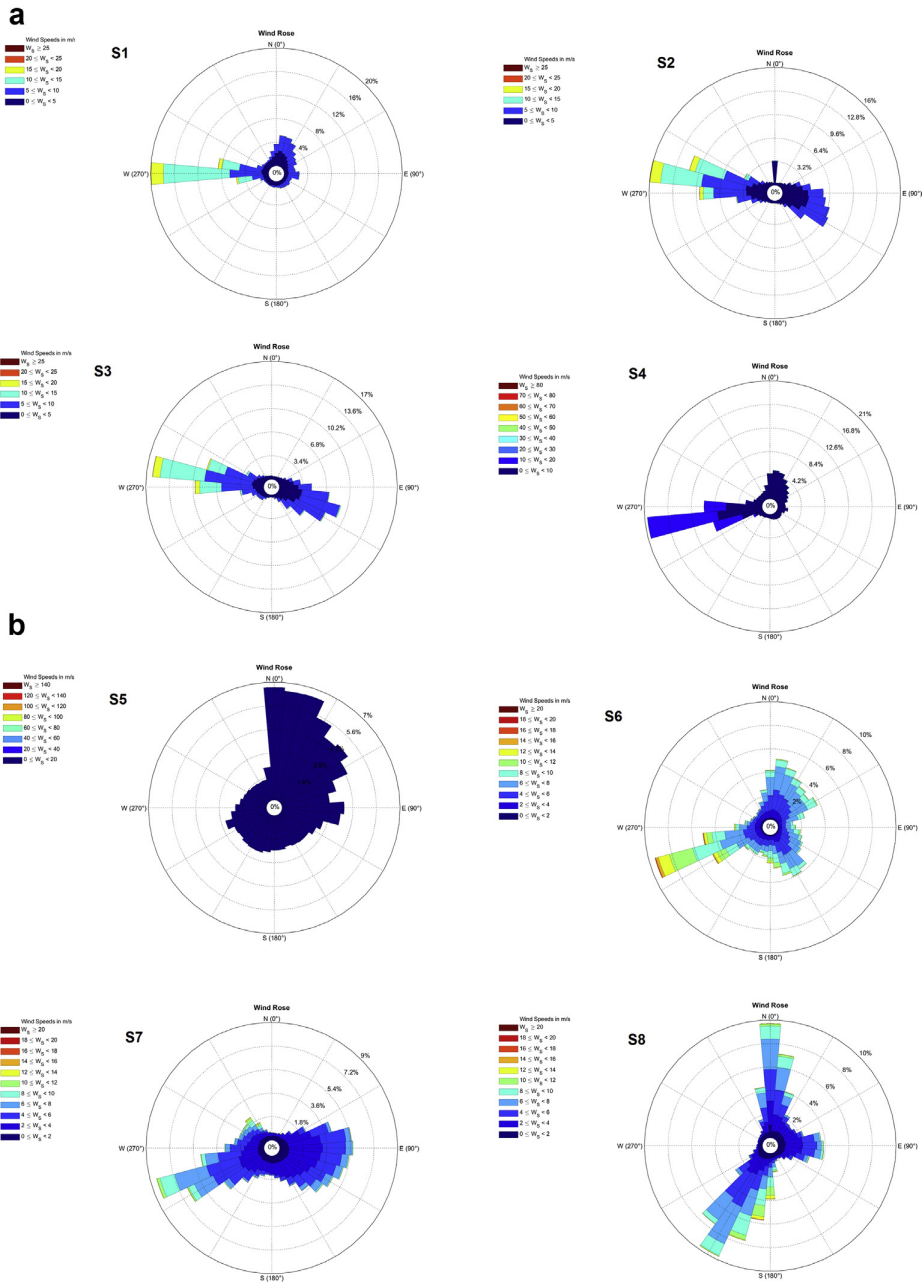

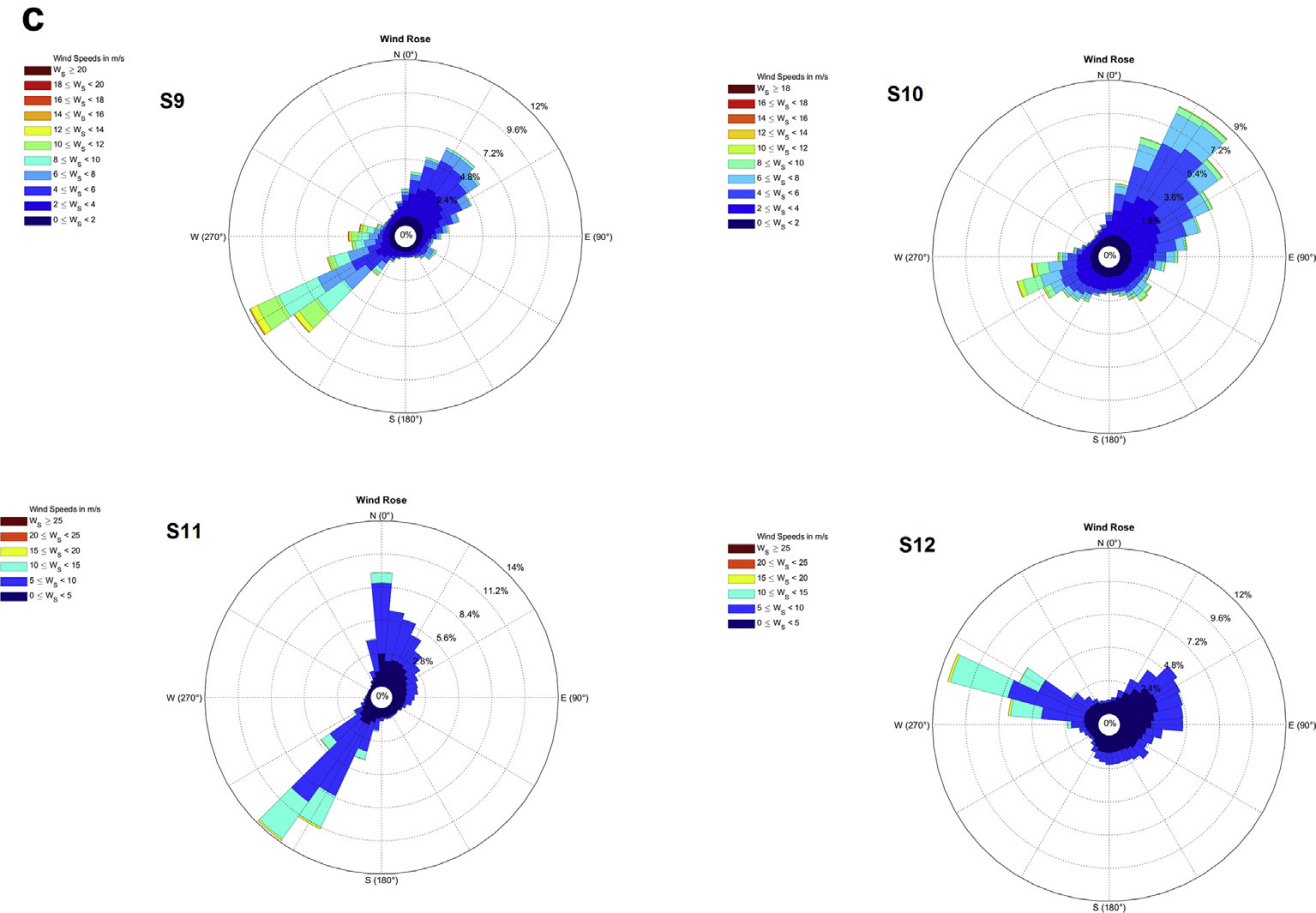
a, b and c represents the annual wind direction and speed of the each site with wind frequency distribution.

The wind data monitoring station was selected from National Institute of Wind energy (NIWE) web portal for long term monitoring of wind data such as wind speed, wind direction, temperature and pressure (Fig. 1). The raw data can be downloaded as only text file. Those text file data is converted into excel file and the required data set such as wind speed, wind direction is separated as given as supplementary material with this article for 12 locations from S1 to S12 as Dataset1.xlsx–Dataset12.xlsx. Each Dataset file consist of data of a particular location for 3 years (2014, 2015 and 2016) for every 10 minutes. These data set in excel file consist of wind speed measured from two instrument one placed at north direction and one at south direction. For wind direction accuracy, the average of these two values are taken into account for the calculation of Wind Power Density (WPD). Fig. 2 represents the annual wind direction and speed of each location with wind frequency distribution. Table 1 represents the average wind direction of the each location, standard deviation (SD), wind power density and uncertainty of the data set for the period of 2014–2016. Table 2 shows the wind power density class for 100 m altitude data.

**Table 1:** Location wise mean monthly wind power density, standard deviation (SD) and uncertainty values.

Station name		Month	Wind power density (W/m^2^)								
			2014			2015			2016		
			Mean	SD	Uncertainty	Mean	SD	Uncertainty	Mean	SD	Uncertainty
S1		January	218.53	151.98	2.27	156.39	129.27	1.93	172.33	131.48	1.97
		Feburary	148.41	130.27	2.05	96.45	119.21	1.88	163.43	148.65	2.30
		March	171.04	162.66	2.43	99.41	100.05	1.52	95.41	107.98	1.62
		April	84.10	102.25	1.56	68.05	105.59	1.61	88.31	109.78	1.67
		May	192.25	225.07	3.37	15.87	91.36	1.37	364.83	472.13	7.07
		June	1385.42	1021.84	15.55	655.65	734.02	11.17	932.81	675.56	10.28
		July	1720.36	960.17	14.37	1014.86	709.11	10.63	969.17	852.97	12.77
		August	796.01	884.83	13.24	698.84	580.27	8.68	894.97	605.75	9.07
		September	661.46	702.39	10.69	397.33	526.26	8.01	755.16	504.24	7.67
		October	15.92	30.41	0.46	112.65	161.32	2.41	200.69	293.31	4.39
		November	17.87	25.78	0.39	81.97	97.75	1.49	85.42	96.29	1.47
		December	150.80	130.23	1.95	174.04	140.60	2.10	162.64	188.13	2.82
S2		January	115.82	115.91	1.73	111.02	107.89	1.61	102.68	96.56	1.45
		Feburary	122.93	129.65	2.04	87.62	108.17	1.70	96.85	106.82	1.69
		March	151.79	134.73	2.02	51.64	82.63	1.24	2.66	7.83	0.12
		April	68.30	103.63	1.58	50.97	99.32	1.51	46.68	76.28	1.16
		May	111.00	151.03	2.37	113.14	150.50	2.25	223.00	293.42	4.39
		June	868.81	822.82	12.52	544.96	767.57	11.68	1026.13	1057.22	16.09
		July	1609.8	1044.31	15.63	887.86	752.27	11.26	922.32	1113.57	16.67
		August	600.83	739.34	11.066	530.24	525.71	7.87	390.04	575.46	8.61
		September	479.28	572.33	8.709	NA	NA	NA	661.08	573.35	8.72
		October	68.355	122.131	1.828	NA	NA	NA	139.56	200.74	3.00
		November	46.848	62.901	0.957	57.71	101.12	1.54	71.94	96.22	1.46
		December	58.109	69.464	1.040	57.32	63.08	0.94	98.01	122.98	1.86
S3		January	172.41	147.66	2.21	158.01	143.72	2.15	144.63	124.88	1.87
		Feburary	191.40	195.92	3.09	158.15	148.52	2.34	166.00	156.70	2.42
		March	186.91	167.26	2.50	144.03	164.22	2.46	125.92	150.23	2.25
		April	101.49	135.54	2.06	78.43	133.30	2.03	88.62	138.87	2.11
		May	185.57	215.01	3.22	147.89	232.54	3.48	239.90	295.80	4.43
		June	811.30	707.78	10.77	649.00	855.48	13.02	698.19	651.34	9.91
		July	1457.35	1014.15	15.18	761.88	547.73	8.33	924.01	1005.83	15.05
		August	644.71	694.75	10.40	454.25	389.74	5.93	793.25	640.57	9.59
		September	482.44	498.80	7.59	278.20	399.49	6.08	520.47	340.55	5.18
		October	78.11	120.08	1.80	70.87	87.58	1.31	148.27	198.08	2.96
		November	70.20	82.00	1.25	82.80	116.79	1.78	116.99	118.94	1.94
		December	95.64	104.90	1.57	88.62	103.19	1.54	58.44	88.93	1.70
S4		January	139.01	127.38	2.06	90.00	94.75	1.42	106.38	112.03	1.68
		Feburary	89.28	101.84	1.60	98.28	102.35	1.61	107.35	133.70	2.07
		March	118.34	145.68	2.18	66.12	73.51	1.10	66.03	89.23	1.34
		April	74.96	143.21	2.20	292.77	385.81	5.78	86.24	128.66	1.96
		May	304.83	338.01	5.18	292.77	385.81	5.78	464.36	588.75	8.81
		June	1485.06	1073.18	16.33	888.84	814.33	12.39	901.49	624.92	9.52
		July	985.97	548.43	16.43	957.90	640.21	9.59	942.35	804.13	12.04
		August	909.11	835.22	12.50	751.02	612.95	9.18	812.73	530.75	7.94
		September	692.95	658.69	10.02	477.06	564.12	8.59	742.05	465.38	7.08
		October	166.78	361.03	5.40	161.28	262.44	3.93	295.49	3578.05	53.57
		November	83.33	105.16	1.60	3126.32	9042.14	144.13	NA	NA	NA
		December	90.94	107.10	1.60	106.89	161.70	2.42	NA	NA	NA
S5		January	181.12	90.90	82.25	122.02	85.27	1.28	145.14	91.70	1.37
		Feburary	120.32	111.45	1.76	130.85	97.04	1.54	116.37	89.14	1.38
		March	116.28	99.22	116.66	92.85	96.68	1.45	90.25	98.34	1.47
		April	69.89	81.04	1.23	61.09	90.33	1.39	64.43	77.77	2.45
		May	69.66	126.41	1.89	57.74	105.07	1.57	NA	NA	NA
		June	81.88	120.62	1.84	36.78	46.46	0.96	NA	NA	NA
		July	101.43	122.27	124.24	281.31	18068.86	272.52	NA	NA	NA
		August	81.89	140.69	2.11	52.30	80.49	1.59	NA	NA	NA
		September	59.51	103.51	73.56	85.67	108.93	1.66	NA	NA	NA
		October	50.70	64.78	0.97	63.46	63.42	0.95	NA	NA	NA
		November	128.72	121.47	1.86	108.34	106.42	1.62	NA	NA	NA
		December	159.08	112.98	1.69	159.25	116.11	1.74	NA	NA	NA
S6		January	266.99	149.65	2.24	191.10	134.86	2.02	219.76	131.67	1.97
		Feburary	192.62	158.10	2.49	213.21	157.42	2.48	192.06	143.50	2.22
		March	193.41	153.59	2.30	140.93	137.52	2.09	140.35	143.09	2.14
		April	145.29	158.76	2.42	127.22	164.97	2.51	172.19	185.12	2.82
		May	189.63	238.66	3.57	165.71	227.46	3.40	177.43	192.92	2.89
		June	481.79	477.40	7.26	445.33	495.45	7.54	364.17	347.21	5.28
		July	565.42	487.55	7.30	303.55	311.19	4.66	367.30	488.84	7.32
		August	343.30	410.21	6.14	258.11	263.19	3.94	319.54	325.94	5.59
		September	314.38	390.21	5.94	319.54	348.90	5.31	232.18	246.40	4.53
		October	87.66	115.56	1.73	127.94	165.44	2.48	122.95	147.66	2.21
		November	145.12	131.58	2.00	110.08	101.55	1.54	134.82	113.35	1.72
		December	209.90	160.68	2.40	207.17	157.54	2.36	172.84	140.70	2.11
S7		January	52.81	68.58	1.03	29.55	41.81	0.63	34.76	50.64	0.76
		Feburary	51.25	70.64	1.11	46.94	70.44	1.11	49.53	63.98	0.99
		March	72.06	98.71	1.48	43.00	77.20	1.17	55.84	65.42	0.98
		April	68.85	105.59	1.61	54.89	156.34	2.38	68.99	96.12	2.31
		May	128.99	177.34	2.65	146.09	266.58	3.99	NA	NA	NA
		June	178.77	235.92	3.59	120.52	182.07	2.77	NA	NA	NA
		July	123.74	202.40	3.03	134.34	185.16	2.77	NA	NA	NA
		August	92.81	125.94	1.88	124.83	195.18	2.92	NA	NA	NA
		September	101.76	149.08	2.27	112.63	181.53	2.76	NA	NA	NA
		October	45.88	100.19	1.50	57.55	122.04	1.83	NA	NA	NA
		November	34.24	45.93	0.70	54.48	95.55	1.45	NA	NA	NA
		December	29.00	47.74	0.71	35.76	56.69	0.85	NA	NA	NA
S8		January	192.32	121.89	6.69	63.82	90.43	1.36	116.82	152.34	2.28
		Feburary	80.23	111.63	1.76	109.39	131.31	2.07	72.08	99.51	1.54
		March	64.71	97.97	1.47	53.37	90.32	1.35	58.61	103.17	1.54
		April	66.46	153.17	2.33	48.06	115.74	1.76	48.71	92.86	2.92
		May	132.59	195.01	2.92	115.98	161.80	2.42	NA	NA	NA
		June	361.75	381.87	5.81	200.82	253.86	3.86	NA	NA	NA
		July	364.64	366.22	5.48	239.33	220.51	3.30	NA	NA	NA
		August	228.22	262.30	3.93	194.25	186.28	2.79	NA	NA	NA
		September	188.06	223.05	3.39	135.03	186.87	2.84	NA	NA	NA
		October	69.80	116.06	1.74	83.96	123.88	1.85	NA	NA	NA
		November	114.48	159.08	2.42	78.77	130.26	1.98	NA	NA	NA
		December	141.57	155.52	2.33	157.17	186.00	2.78	NA	NA	NA
S9		January	109.85	106.17	2.33	57.28	69.35	1.04	63.10	67.07	1.00
		Feburary	77.17	92.22	1.45	76.19	84.15	1.33	83.66	102.24	1.58
		March	93.09	107.13	1.60	52.29	78.69	1.18	59.20	99.42	1.49
		April	20.79	64.59	0.98	49.21	111.73	1.70	NA	NA	NA
		May	28.77	34.17	0.51	232.25	316.47	4.74	NA	NA	NA
		June	427.76	398.68	6.07	486.80	411.10	6.25	NA	NA	NA
		July	502.11	474.63	7.10	310.01	259.60	3.89	NA	NA	NA
		August	355.20	346.19	5.18	277.74	248.61	3.72	NA	NA	NA
		September	299.23	316.49	4.82	290.40	330.78	5.03	NA	NA	NA
		October	80.54	163.25	2.44	148.13	257.83	3.86	NA	NA	NA
		November	60.16	80.43	1.22	57.96	111.81	1.70	NA	NA	NA
		December	51.51	75.34	1.13	192.68	140.22	2.10	NA	NA	NA
S10		January	139.40	115.00	1.72	83.37	85.16	1.27	103.79	112.23	1.68
		Feburary	82.98	95.56	1.50	105.67	105.48	0.53	98.55	104.45	1.62
		March	91.89	107.63	1.61	60.34	84.88	1.29	62.79	104.51	1.56
		April	70.44	156.00	2.37	62.99	135.67	2.06	59.68	123.74	3.11
		May	68.08	171.17	2.56	39.97	98.14	1.47	NA	NA	NA
		June	125.22	191.19	2.91	154.03	263.34	4.01	NA	NA	NA
		July	137.01	196.23	2.94	91.23	152.07	2.28	NA	NA	NA
		August	121.24	182.75	2.74	81.45	139.03	2.08	NA	NA	NA
		September	99.43	170.95	2.60	113.73	182.69	2.78	NA	NA	NA
		October	43.91	75.72	1.13	47.30	80.94	1.21	NA	NA	NA
		November	91.74	110.47	1.68	58.22	74.58	1.13	NA	NA	NA
		December	108.50	124.54	1.86	146.58	154.38	2.31	NA	NA	NA
S11		January	219.60	150.78	12.19	176.18	75.85	3.36	142.26	96.89	1.45
		Feburary	143.49	134.29	2.11	144.68	114.97	1.81	120.83	103.67	1.60
		March	131.59	123.95	1.86	103.41	112.35	1.68	79.29	97.31	1.46
		April	74.10	107.12	1.63	63.72	131.72	2.00	64.00	97.40	1.48
		May	102.85	239.72	7.06	144.61	204.96	3.07	219.80	258.23	3.86
		June	705.69	505.67	8.21	267.95	577.23	8.78	470.87	337.97	5.14
		July	821.05	523.15	7.83	331.78	376.33	5.63	478.42	514.59	7.70
		August	416.71	430.01	6.44	301.45	261.55	3.92	387.64	294.09	4.41
		September	460.66	320.73	5.98	220.71	268.57	4.09	340.62	238.91	3.63
		October	NA	NA	NA	70.11	90.79	1.36	102.02	138.34	2.07
		November	139.39	134.52	4.95	142.45	299.28	4.55	138.93	114.41	1.74
		December	147.74	127.62	3.07	166.20	147.86	2.21	166.95	157.61	2.36
S12		January	NA	NA	NA	96.60	88.80	1.33	98.30	79.55	1.19
		Feburary	NA	NA	NA	99.36	95.24	1.50	87.19	98.47	1.52
		March	102.17	105.00	1.90	79.38	99.78	1.49	73.51	96.50	1.44
		April	79.17	116.63	1.77	76.44	122.74	1.87	73.89	93.60	3.49
		May	145.63	222.93	3.34	105.12	233.70	3.50	NA	NA	NA
		June	596.61	519.17	7.90	366.51	547.34	8.33	NA	NA	NA
		July	843.70	605.44	9.06	548.92	468.99	7.02	NA	NA	NA
		August	333.75	393.59	5.89	340.51	331.03	4.96	NA	NA	NA
		September	270.01	325.01	4.94	180.81	254.06	3.87	NA	NA	NA
		October	71.18	109.09	1.63	55.84	106.44	1.59	NA	NA	NA
		November	88.96	86.14	1.31	108.55	157.39	2.39	NA	NA	NA
		December	104.19	85.26	1.28	96.88	90.08	1.35	NA	NA	NA

NA – Data not available; SD – Standard Deviation.

**Table 2:** Wind power class with respect to height (100 m) in India.

Wind power class	Height of the monitoring instruments – 100 m	
	Wind power density (w/m^2^)	Mean annual wind speed (m/s)
1	0–180	0–5.4
2	180–210	5.4–5.6
3	210–250	5.6–6.0
4	250–300	6.0–6.4
5	300–350	6.4–6.7
6	350–400	6.7–7.0
7	<400	<7.0

## 2 Experimental design, materials, and methods

The wind speed was monitored using field cup anemometer (Instrument Make - Adolf Thies GmbH&Co. KG, Germany). The wind direction was recorded using Thies compact TMR wind vane (KINTECH Engineering). The power density was calculated from wind direction and speed of the each monitoring location. The monitoring instrumental setup was installed at 100 m altitude in 12 monitoring location for continuous data collection for every 10 minutes. The large volume of wind data was processed using Microsoft Excel 2007 software package. The meteorological data processing, wind rose diagram and statistical analysis were carried out using MATLAB 2016 software package. Wind power density was calculated using meteorological parameters such as wind speed distribution, air density and cube of wind speed. The available wind potential ($P_{a}$) per unit area is perpendicular to the wind stream. According to Rehman et al., 1994 [1], the kinetic energy flux is expressed as follows:

(1) $$P_{a}=0.5\rho v^{3}$$

In the above equation, v is the wind speed (in m/s); ρ is the air density (in kg/m^3^); and $P_{a}$ is the theoretically available wind potential (in w/m^2^). The generation of wind power depend on the wind energy conversation system and intensity of the wind in particular location. According to the above concept, approximately, 40% of the available wind power must be reached at the maximum. According to Betz's [2] limit, the maximum extractable power $P_{max}$ from a system working at its optimum efficiency is limited by a power coefficient (0.593; [2]). This capacity factor makes the maximum extractable power approximately 59.3% of the theoretical wind power [3].

(2) $$P_{\max}=\frac{0.593}{2}\rho v^{3}$$

The monitoring sites were classified based on the geographical locations and the distance from the Western ghats pass (gaps). Among the twelve monitoring locations, five locations falls in Aralvaimozhi and Senkottah pass sector (L.No S1, S4, S6, S9 and S10). The remaining seven locations falling under Palghat pass sector (L.No S2, S3, S5, S7, S8, S11 and S12). The calculated average wind speed of monitoring locations (from 2014 to 2016) is ordered as: S4 > S1 > S3 >S6 > S11 > S2 > S12 > S9 > S8 > S5 > S10 > S7. The wind energy potential (W/m2) of the monitoring locations is proportional to the wind speed, which follows the above mentioned order. According to Poje and Cividini [4], the wind energy potential of the each sites were classified based on the wind power class. Among all the monitoring locations, station 1, 2, 3, 4 were falling between wind power class 5 to 7 (Table 2). The other monitoring locations were falling between wind power class 1 to 3. The outcome of the wind power class clearly reveals that the geomorphological features like altitude of the monitoring locations and Aralvaimozhi, Senkottah and Palaghat gaps significantly affects the wind power density of the individual sites. The above assumption was observed through the regular monitoring of the wind speed, direction and power density of the monitoring stations.

## Funding

This research did not receive any specific grant from funding agencies in the public, commercial, or not-for-profit sectors.

## Appendix A Supplementary data

Dataset1 Three year Data set of Location S1 Dataset1

Dataset2 Three year Data set of Location S2 Dataset2

Dataset3 Three year Data set of Location S3 Dataset3

Dataset4 Three year Data set of Location S4 Dataset4

Dataset5 Three year Data set of Location S5 Dataset5

Dataset6 Three year Data set of Location S6 Dataset6

Dataset7 Three year Data set of Location S7 Dataset7

Dataset8 Three year Data set of Location S8 Dataset8

Dataset9 Three year Data set of Location S9 Dataset9

Dataset10 Three year Data set of Location S10 Dataset10

Dataset11 Three year Data set of Location S11 Dataset11

Dataset12 Three year Data set of Location S12 Dataset12

## References

[1] Rehman S., Halawani T.O., Husain T. (1994). Weibull parameters for wind speed distribution in Saudi Arabia. Sol. Energy.

[2] Betz A. (1966). Introduction to the Theory of Flow Machines.

[3] Mohandes M., Rehman S., Halawani T.O. (1998). A neural network approach for wind speed prediction. Renew. Energy.

[4] Poje D., Cividini B. (1988). Assessment of wind energy potential in Croatia. Sol. Energy.

